# Supplementary material for: Self-organizing neuromorphic nanowire networks as stochastic dynamical systems
Source: Nat Commun. 2025 Apr 13;16:3509. doi: 10.1038/s41467-025-58741-2 (PMC11994789; doi:10.1038/s41467-025-58741-2)
Supplement: Supplementary file 1 — Supplementary Information [file 41467_2025_58741_MOESM1_ESM.pdf]

Self-organizing neuromorphic nanowire networks as stochastic dynamical systems

Gianluca Milano[1*], *Fabio Michieletti*[2], *Davide Pilati*[1,2], *Carlo Ricciardi*,[2] *Enrique Miranda*[3]

[1]Advanced Materials Metrology and Life Sciences Division, INRiM (Istituto Nazionale di Ricerca Metrologica), Strada delle Cacce 91, 10135 Torino, Italy.

[2]Department of Applied Science and Technology, Politecnico di Torino, C.so Duca degli Abruzzi 24, 10129 Torino, Italy.

[3]Departament d'Enginyeria Electrònica, Universitat Autònoma de Barcelona (UAB), 08193 Cerdanyola del Vallès, Spain

Email: g.milano@inrim.it

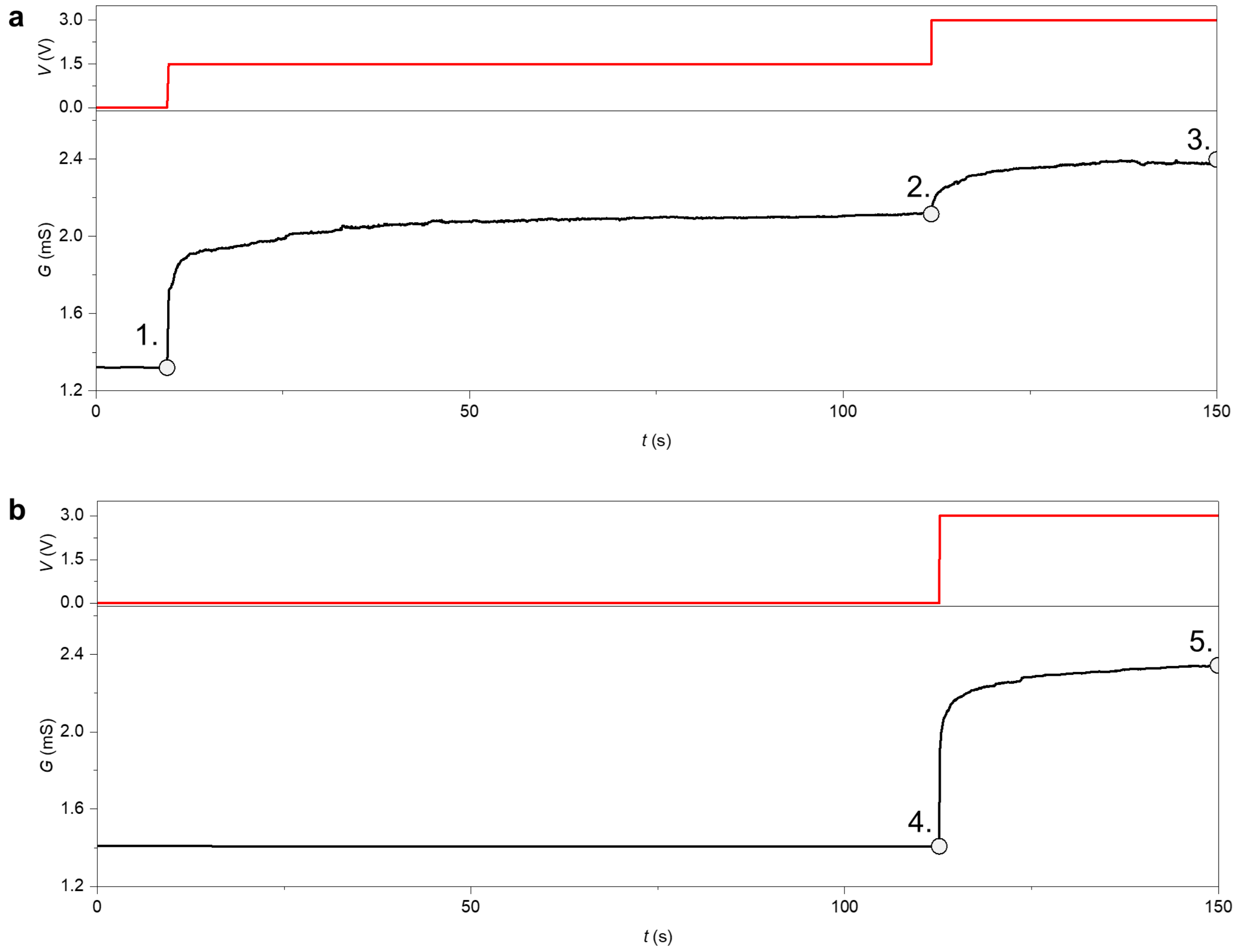

Supplementary Figure 1 | Evolution of the system towards the steady state. a. Experimental **e**volution of the system in terms of conductance when progressively stimulated with 1.5 V and 3 V bias voltages**.** When stimulated with 1.5 V, the system evolves from an initial condition (state 1) towards a new equilibrium condition (state 2) representing the steady state for the 1.5 V stimulation bias. This new equilibrium condition represents the new initial condition when the system evolves to a new steady state (state 3) when the bias voltage is increased to 3 V. **b.** Experimental evolution of the system when stimulated with 3 V bias voltage. In this case, the system evolves from the initial condition (state 4) towards a new equilibrium condition (state 5). By comparing conductance evolution reported in panel a and b, it is worth noticing that the system tends to the same steady state (out of experimental variability) when driven by the same applied voltage (compare state 3 and 5), even if the evolution starts form a different initial condition (compare state 2 and 3).

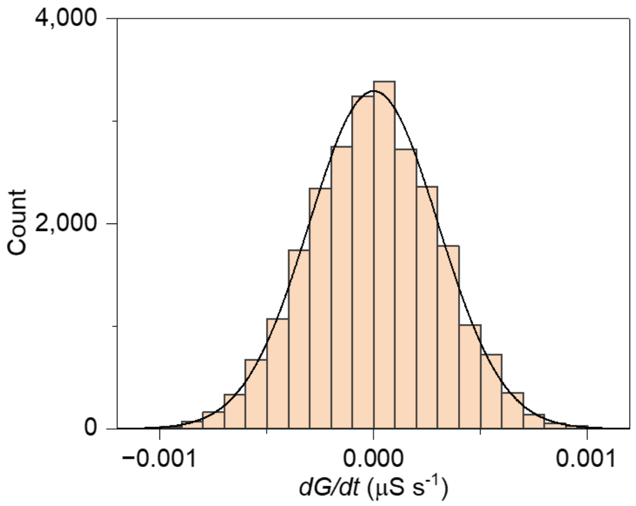

Supplementary Figure 2 | Experimental noise observed in a resistor. Histogram of dG/dt fitted with a Gaussian distribution (black like) obtained by evaluating the time trace of conductance (~ 15,000 s) of a discrete resistor with a resistance value of 27 kΩ comparable to the mean value of resistance observed in the stationary state of the NW network experimentally analyzed in Figure 3. Note that the conductance time trace in case of the discrete resistor has been measured by exploiting the same experimental setup and measurement protocol used for the characterization of the NW network. The standard deviation of dG/dt observed in a discrete resistor is ~ $3 \cdot 10^{-10}$ S s^{-1}, two orders of magnitude lower than the standard deviation of dG/dt of ~ $3 \cdot 10^{-8}$ S s^{-1}observed in the Gaussian component of noise of the NW network (please refer to Figure 3e). This observation shows that the Gaussian component of noise observed in NW networks results from the physical substrate and does not rely on the measurement setup and/or measurement scheme.

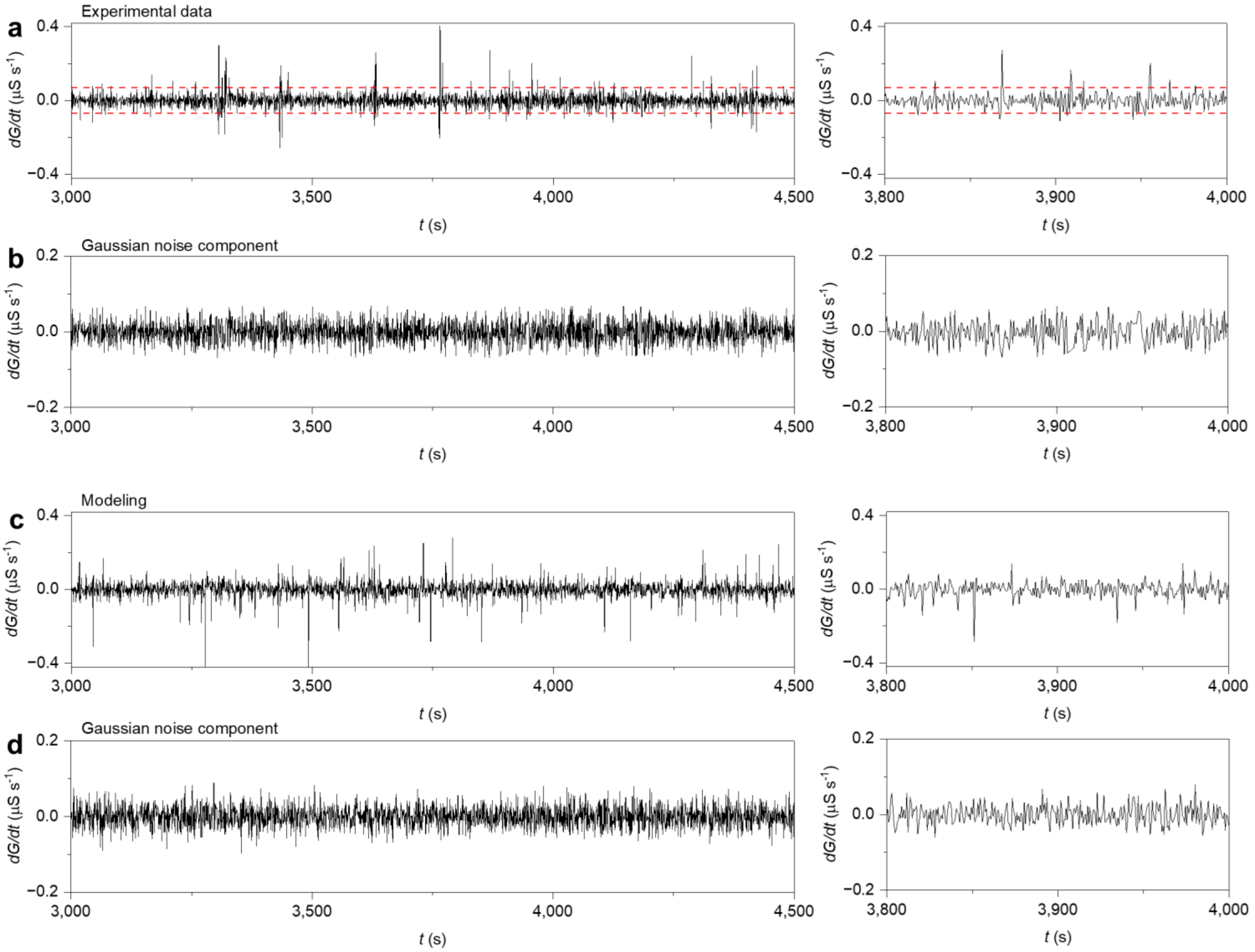

Supplementary Figure 3 | Direct comparison of experimental and modeled changes in the conductance time traces. Comparison on different time scales of **a.** experimental changes in the conductance and **b.** corresponding Gaussian noise component of the experimental time trace with **c.** modeled changes in the conductance and **d.** corresponding Gaussian noise component of the modeled time trace. Data are replotted from Figures 3 and 4, showing good correspondence of modeling results with experimental data on multiple time scales.

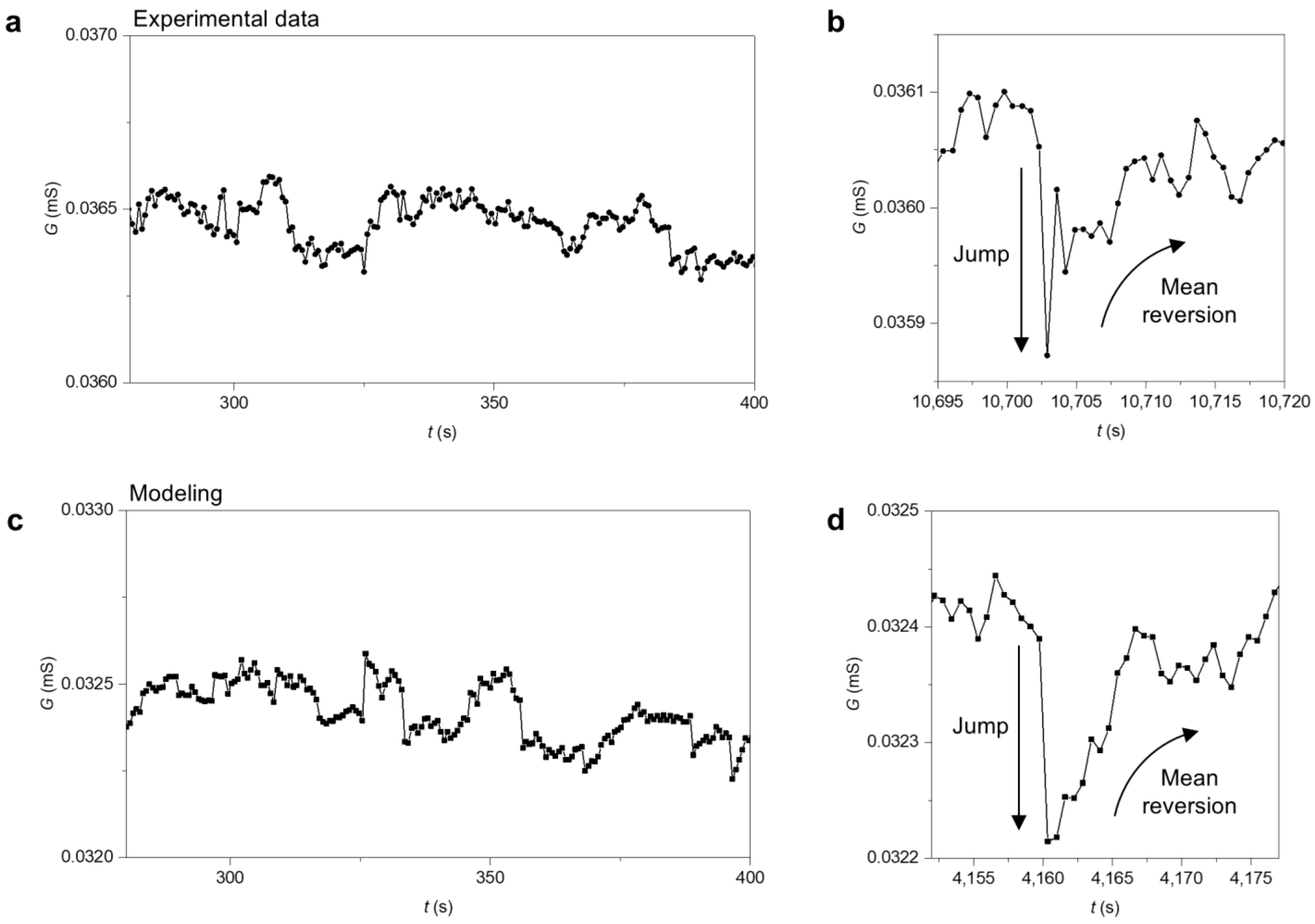

Supplementary Figure 4 | Direct comparison of experimental data and modeling. a. Experimental conductance time trace and **b.** experimental example showing the reversion to the mean effect after a jump in the conductance time trace. **c.** Modeled conductance time trace and **d.** modeled example showing the reversion to the mean effect after a jump in the conductance time trace. Experimental and modeled time traces for a stationary state sustained by an applied constant voltage of 3.6 V are reported. Note that direct visualization of the mean-reverting property even in the modeled conductance time trace (where mean reversion is imposed by the equation) is not straightforward due to the unavoidable co-occurrence of noise and jumps, as can be observed in panel c.

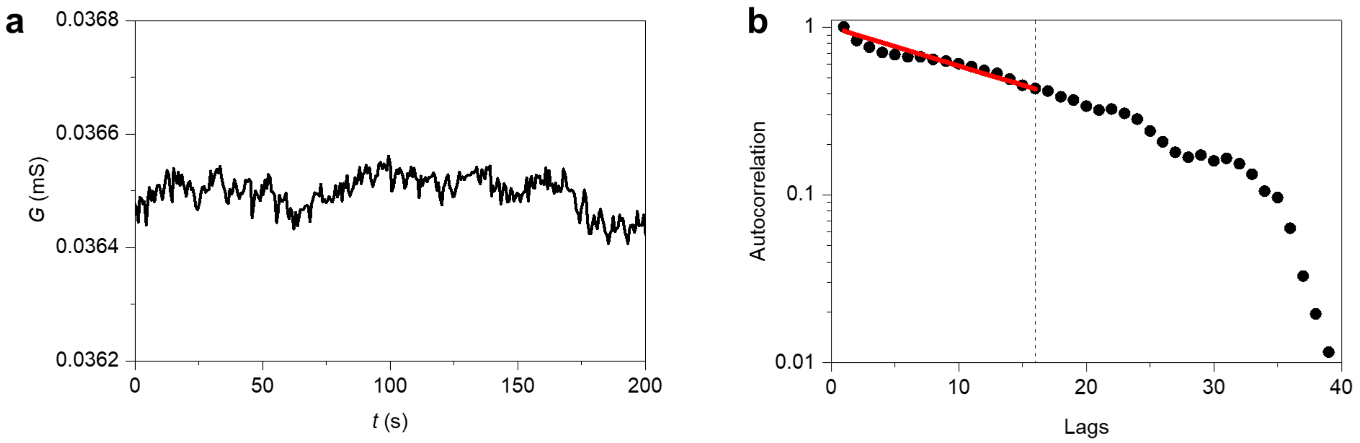

Supplementary Figure 5 | Autocorrelation plot of experimental data acquired in a stationary state. a. Example of an experimental conductance time trace of a network in a stationary state sustained by an applied bias voltage of 3.6 V and b. corresponding autocorrelation plot. The autocorrelation plot, where it is possible to see that autocorrelation decreases as a function of lags, shows that the conductance time trace cannot be simply modeled by superimposing a (uncorrelated) noise to a deterministic trajectory. Indeed, in a stationary state no autocorrelation is expected if deterministic and stochastic dynamics are not coupled (i.e., simply adding noise to a deterministic trajectory in a stationary state does not result in autocorrelation). Note that the exponential trend of the autocorrelation function of the signal as a function of lags (in a stationary state) agrees with the expected autocorrelation (exponential) from an OU process.[1] The fitting of the autocorrelation function (red line) was performed by considering correlations that are significant with a 95 % confidence interval (left side of the vertical dashed line). The lag relies on the sampling rate of the measurements and corresponds to Δt ~ 0.63 s.

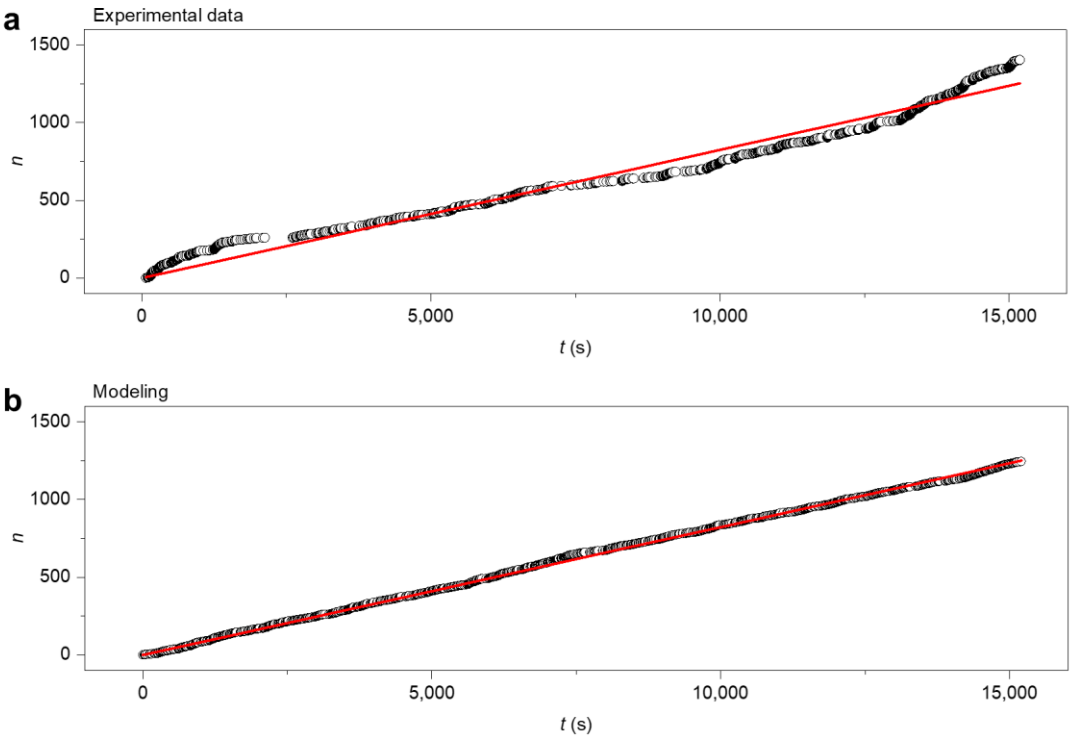

Supplementary Figure 6 | Jump events as a function of time. a. Experimental and **b.** modeling results of jumps events n as a function of time (enlarged view of data reported in Figure 3g and 4g, respectively). Data (circles) are interpolated by a straight line with slope $\lambda \sim 0.082$ events/s (event rate or intensity).

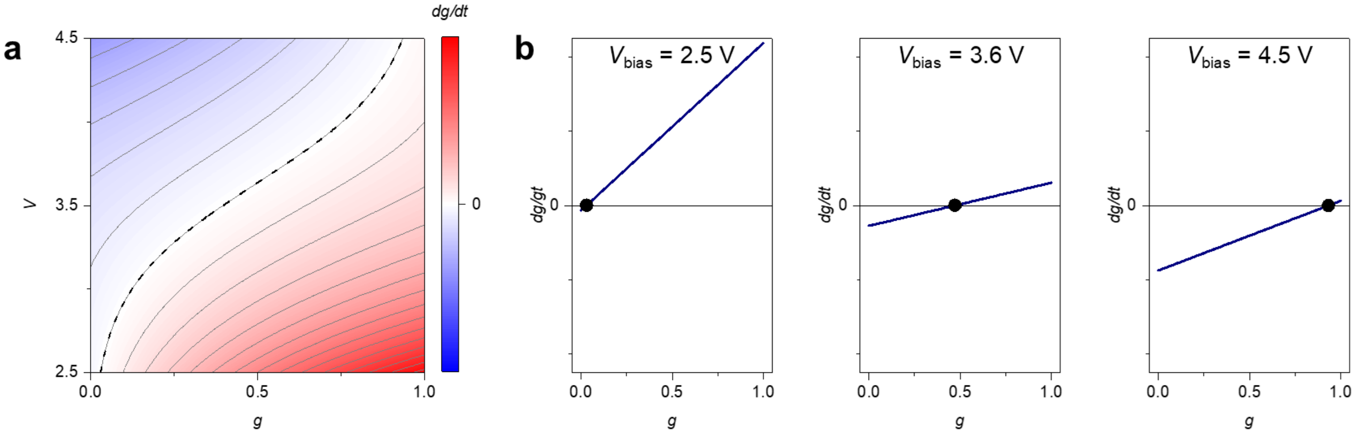

Supplementary Figure 7 | Phase portrait of the nanoscale network dynamical system. a. Landscape of dg/dt as a function of the normalized internal state of the network g and applied voltage V obtained by modeling. The black dashed line represents the curve dg/dt = 0 that corresponds to the asymptotic steady state of the system, where potential function assumes minimum values. **b.** Profiles of dg/dt at fixed voltage bias, where the black dot points out the intersection of the profile with dg/dt = 0 (steady state).

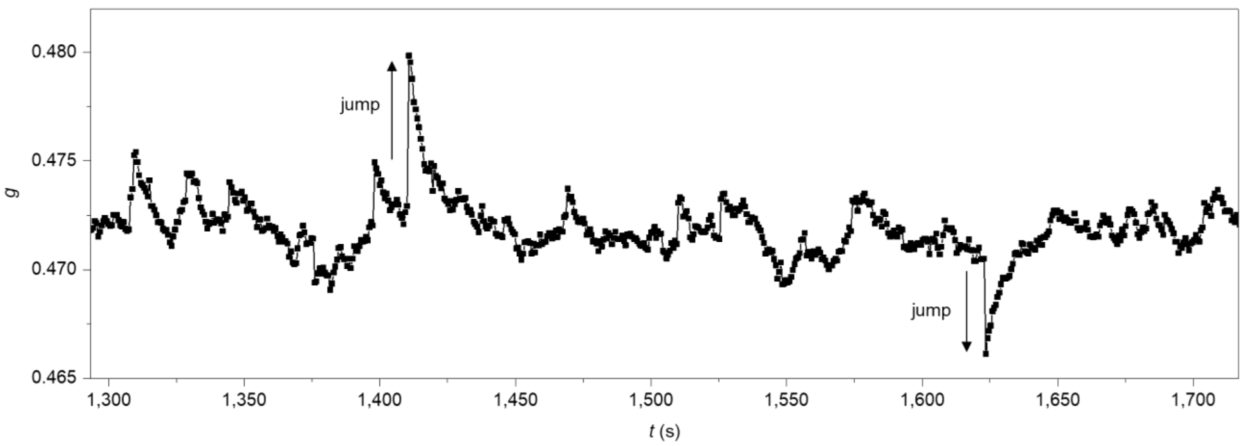

Supplementary Figure 8 | Detail of the evolution of the internal memory state g. Evolution over time of the simulated internal memory state of the stochastic dynamical system in the stationary state sustained by an applied voltage of 3.6 V. The figure represents an enlarged view of the memory state time trace reported in Figure 6c and highlights the presence of up and down conductance jumps.

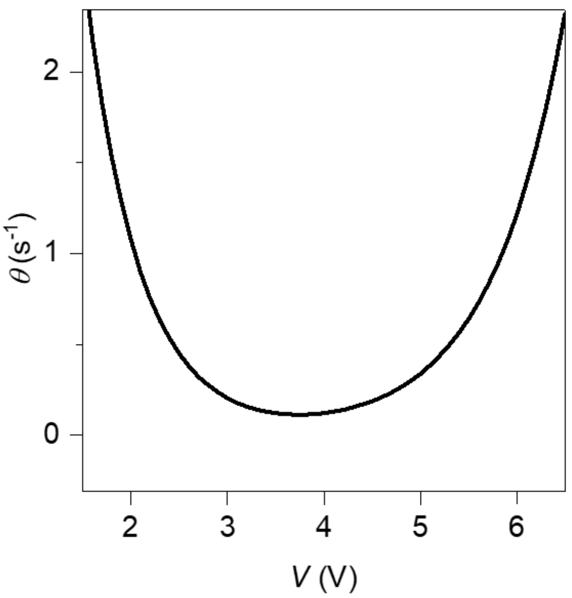

Supplementary Figure 9 | Reversion speed θ of the OU process as a function of the bias voltage. Dependence of the reversion speed θ on the bias voltage (zoomed version of data reported in Figure 2e) obtained from modeling. Higher reversion speeds result in outputs that respond faster to the input, while a lower reversion speed results in an output that respond slower to the input (note that the network response speed relies also on the input signal frequency and amplitude of the signal). Note that the θ dependence on the bias voltage has been obtained by interpolating experimental data reported in Figure 2b with the proposed modeling approach

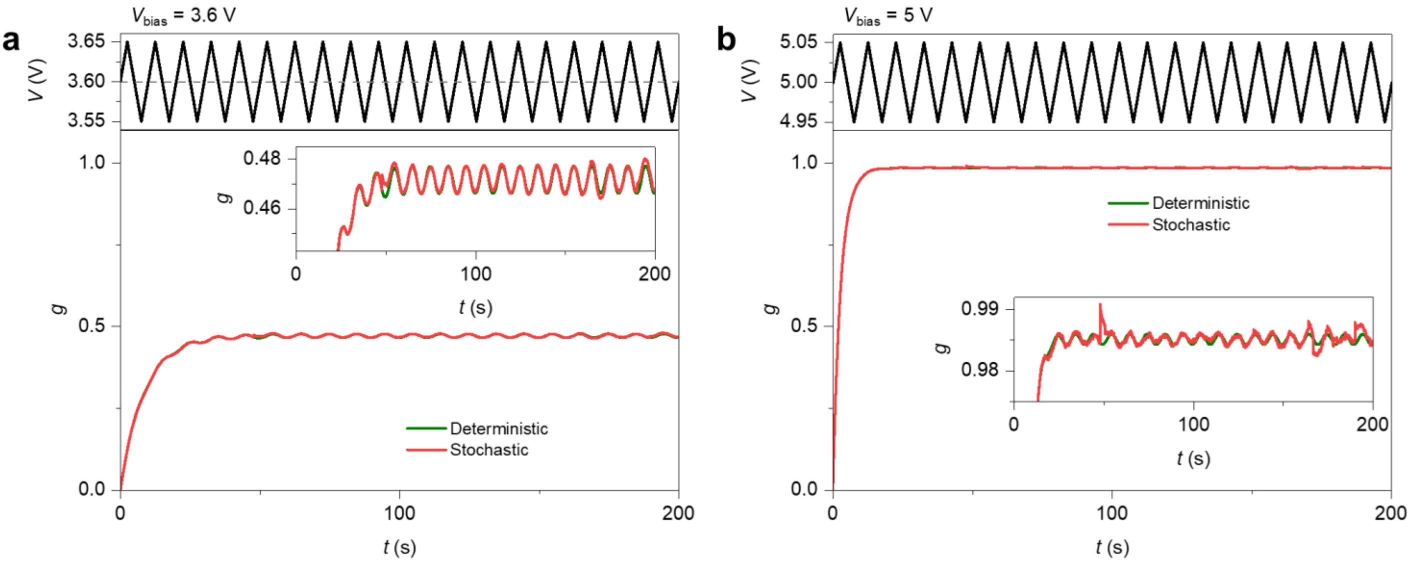

Supplementary Figure 10 | Processing of the input signal through neuromorphic network dynamics – transient dynamics. Deterministic and stochastic evolution of the internal memory state of the NW network g when stimulated with a triangular voltage waveform with amplitude of 50 mV (external signal), while applying a constant bias of **a.** 3.6 V and **b.** 5 V by considering the ground state of the system as initial state. Results are obtained from modeling. As can be observed, transient dynamics are induced by the applied constant bias to drive the system to a fixed-point steady state where it is possible to process the input signal. Insets show an enlarged view of the network response. Note that information processing based on fluctuations around a fixed-point steady state can be performed only after stabilization of the system near the steady state induced by the voltage bias (operating regime). Data reported in Figure 7 are extracted from the last 100 s of network dynamics where transient dynamics related to the constant bias are no longer observed.

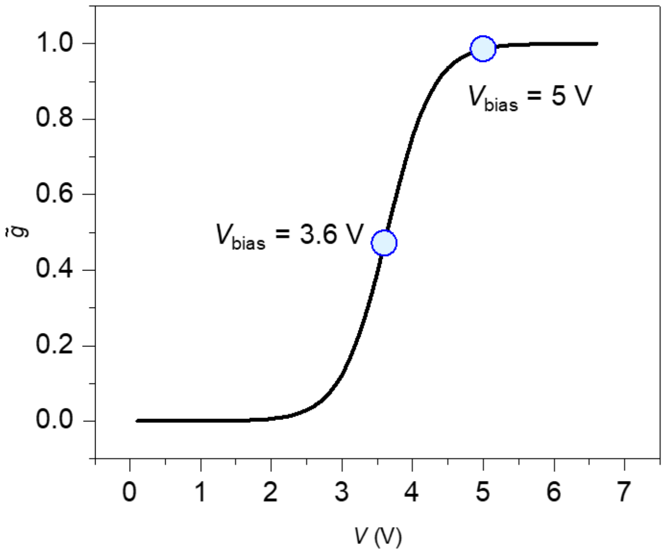

Supplementary Figure 11 | Operating regimes of the network. Evolution of the internal memory stationary steady state $\tilde{g}$ as a function of the applied voltage bias from modeling. Circles represent the fixed-point steady state for the operational regimes discussed in Figure 7. Note that the operating regime sustained by a bias of 3.6 V drives the system close to the center of the steady state sigmoidal-like transition expected in stationary conditions ($\tilde{g}$ ~ 0.5), while the operating regime sustained by a bias of 5 V drives the system to a $\tilde{g}$ value close to 1

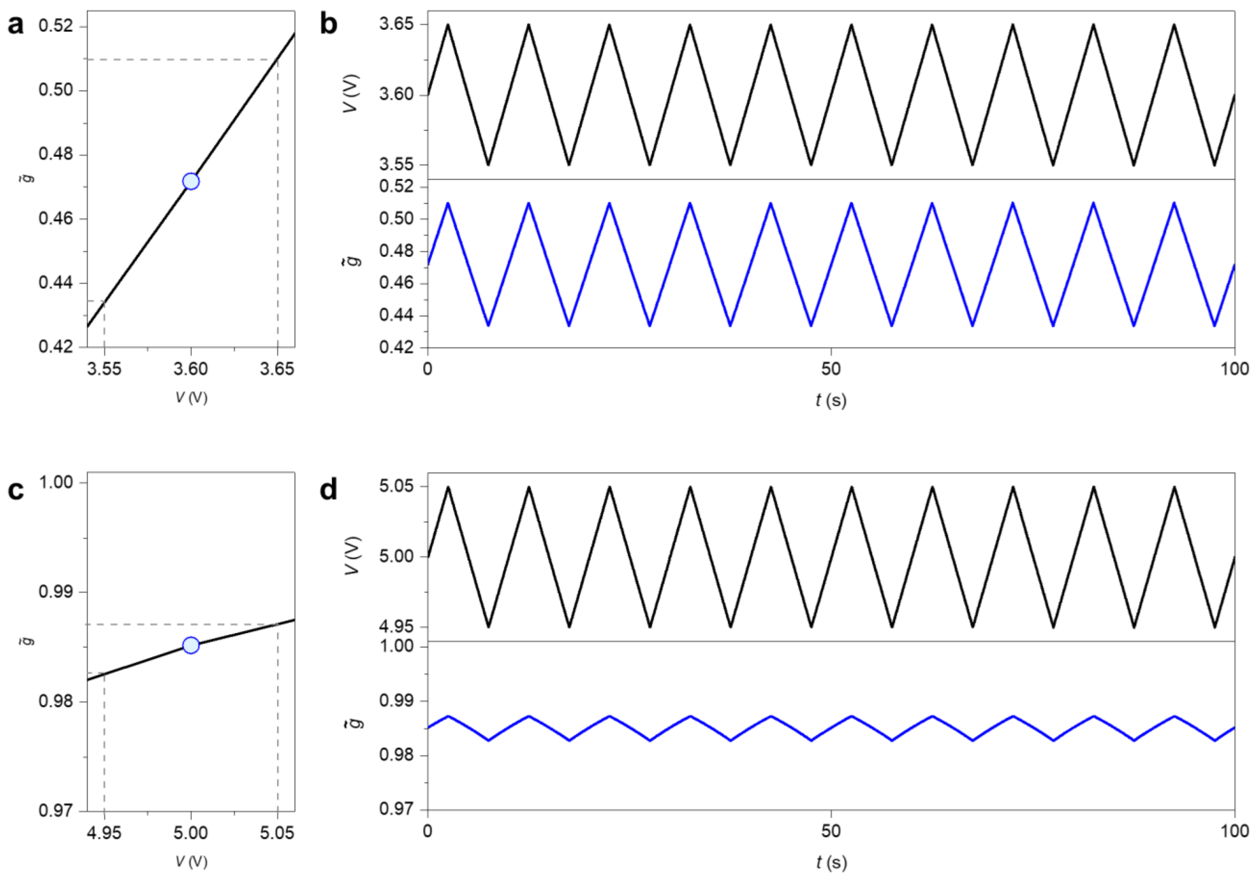

Supplementary Figure 12 | Processing of the input signal through fluctuations of the steady state. a. Steady state of the system $\tilde{g}$ in the operating regime sustained by a constant bias of 3.6 V (blue circle) and range of fluctuations of $\tilde{g}$ when stimulated with a triangular voltage waveform with amplitude of 50 mV obtained by modeling; **b.** corresponding fluctuations over time of the steady state $\tilde{g}$. **c.** Steady state of the system $\tilde{g}$ in the operating regime sustained by a constant bias of 5 V (blue circle) and range of fluctuations of $\tilde{g}$ when stimulated with a triangular voltage waveform with amplitude of 50 mV; **d.** corresponding fluctuations over time of the steady state $\tilde{g}$. Fluctuations of $\tilde{g}$ over time are responsible for transient dynamics that can be exploited for nonlinearly processing of the input signal. Note that the operational regime sustained by a 3.6 V bias results in a larger range of $\tilde{g}$ fluctuations compared to the operational regime sustained by 5 V, enhancing in this case the dynamical range of the system's output.

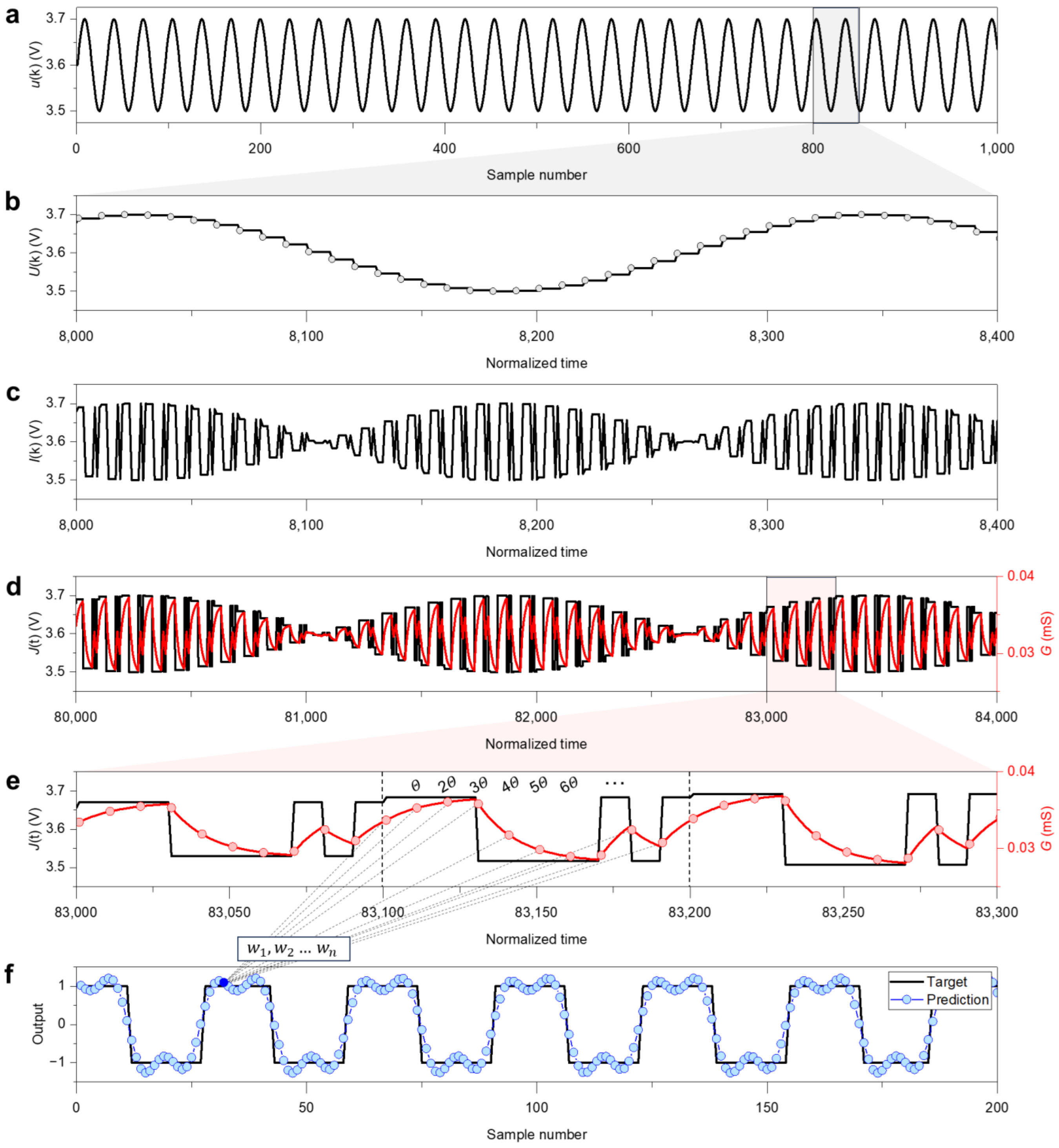

Supplementary Figure 13 | Time-multiplexed reservoir computing implementation based on a single dynamical node. Given an input signal $u(k)$ (panel a), each sample of the input signal $u(k_i)$ is held for N samples (panel b), where N is the number of virtual nodes (panel b). Then, the piecewise signal $U(k)$ is multiplied by a binary mask (panel c), which is the same for each input sample $u(k_i)$ to obtain a masked input signal $I(k)$. Each sample of the masked signal $I(k_i)$ is further held for Θ samples, each of duration Δt (where Δt is the time unit, in our case $\Delta t = 0.63$ s according to

experimental measurements), and then fed into the network as the input signal $J(t)$ (panel d). In this framework, the effect of a single sample $u(k_i)$ of the original input lasts for $\tau = \Theta \times N$. Once $J(t)$ is fed, the network response in terms of conductance is extracted at each virtual node (panel d, zoom in panel e). The linear combination of weighted network responses is then exploited to generate the output of the system, where weights **w** are trained by linear regression. A comparison of the predicted output and target output of the system when transforming the input sine wave to a square wave is reported in panel f.

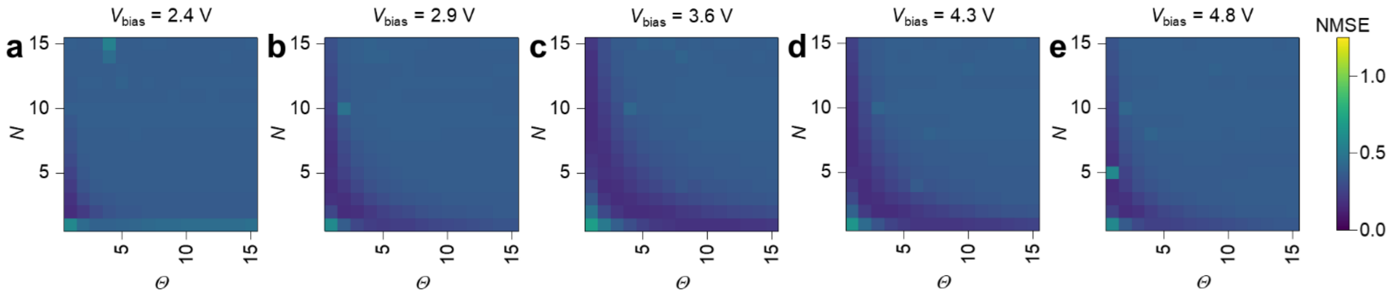

Supplementary Figure 14 | Effect of *N* and Θ on the NARMA task performance depending on the polarization voltage. Colormaps showing NARMA-2 task performances simulated with the deterministic model as a function of polarization voltages of **a.** 2.4 V, **b.** 2.9 V, **c.** 3.6 V ($\tilde{g}$ ~ 0.5), **d.** 4.3 V, and **e.** 4.8 V, by stimulating the network with an input amplitude of 50 mV. A substantial decrease in performance can be observed by considering sets of parameters with high *N* or high Θ values when operating the network away from $\tilde{g}$ ~ 0.5, since in these cases network dynamics are more affected by a reduction of fading memory properties.

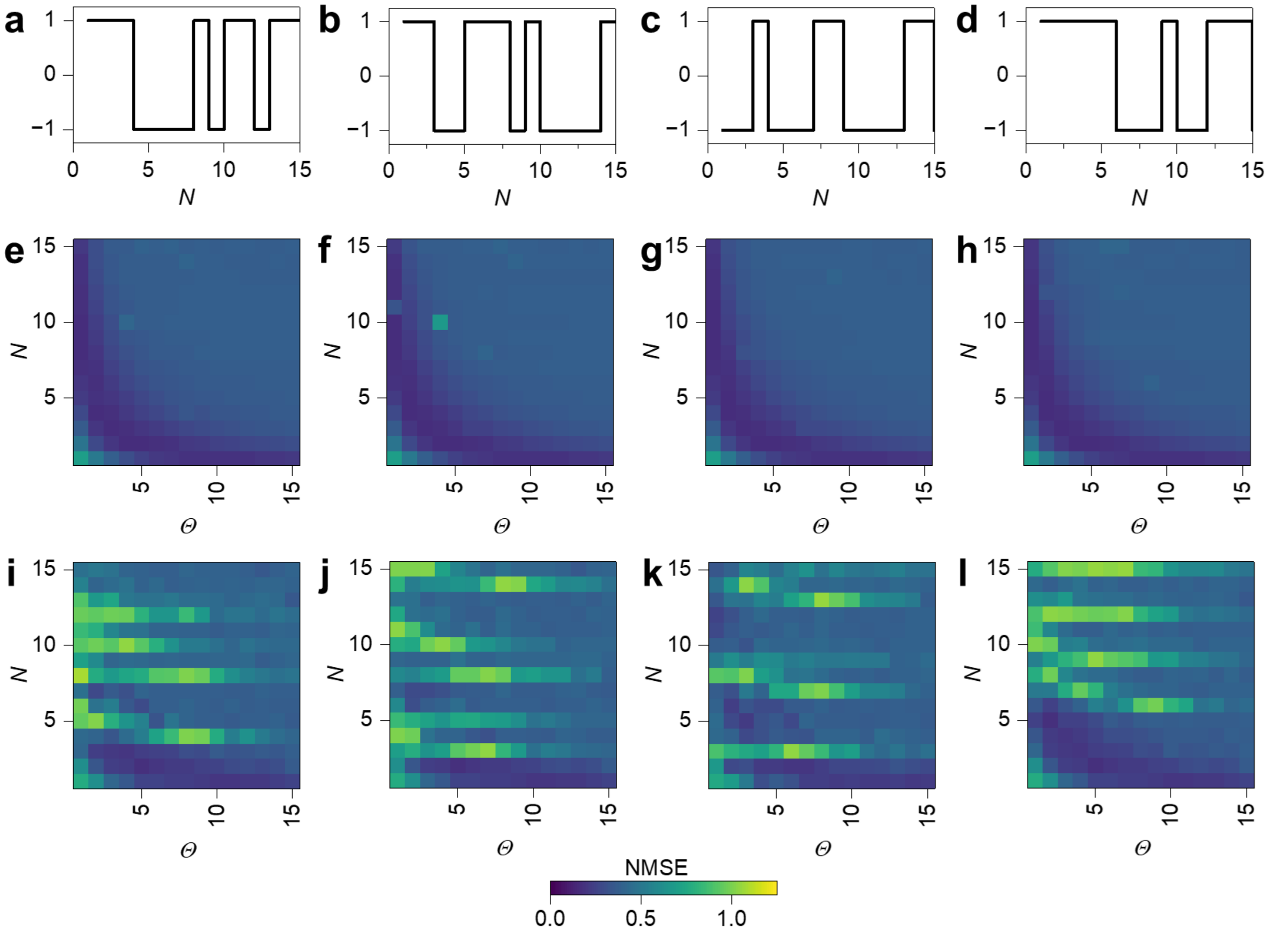

Supplementary Figure 15 | Effect of the masking scheme during time multiplexing on the NARMA-2 task performance. **a-d.** Different masking schemes and corresponding colormaps showing task performances as a function of N and Θ parameters simulated by exploiting the **e-h.** deterministic and **i-l** stochastic model. Results have been obtained with a polarization voltage of 3.6, operating the network at $\tilde{g} \sim 0.5$. While the masking scheme does not substantially affect performance in terms of NMSE in the deterministic case, mask-dependent degradation of performances can be observed for some specific sets of $[N, \Theta]$ parameters. This indicates that the peculiar dynamics of the network induced by the masking scheme combined with the signal input can lead to computing performances of the system that can be less or more affected by stochastic effects.

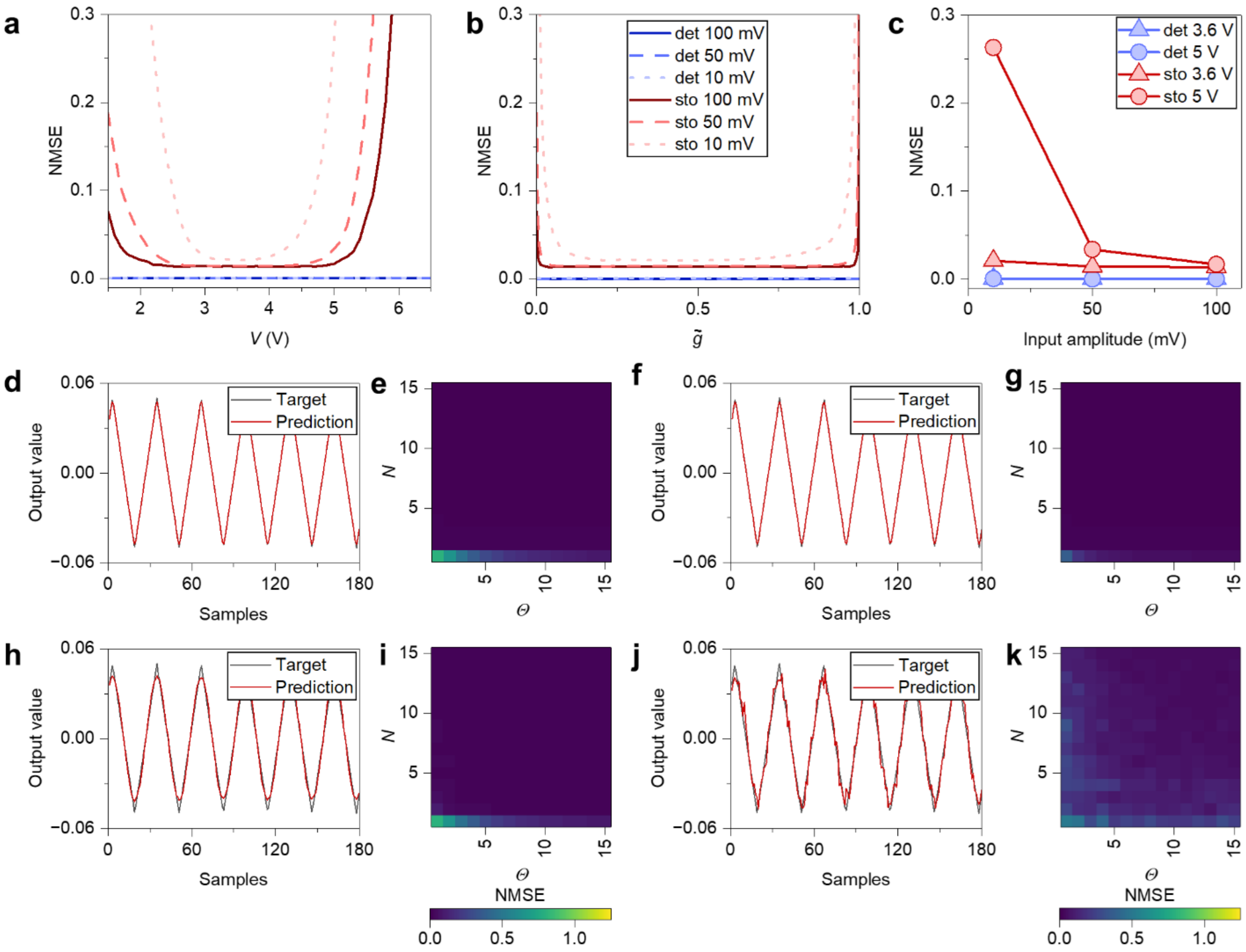

Supplementary Figure 16 | Nonlinear transformation (NLT) task. Simulation results of the sine to triangular waveform NLT task in terms of NMSE as a function of **a.** the bias voltage and **b.** the steady state $\tilde{g}$ by considering deterministic and stochastic dynamics corresponding to different amplitudes of the input signal. **c.** NMSE as a function of the input signal amplitude for deterministic and stochastic dynamics, by considering polarization voltages of 3.6 V ($\tilde{g}$ ~ 0.5) and 5 V ($\tilde{g}$ ~ 0.99). Predictions of the sine to triangular waveform NLT relative to polarization voltages of 3.6 V and 5 V obtained with optimal parameters through deterministic dynamics in panels **d.** and **f.**, respectively ($[N, \Theta]$ in panels d and f are [15,12] and [15,15], respectively). Colormaps showing task performances as a function of N and Θ parameters for polarization voltages of 3.6 V and 5 V in panels **e.** and **g.**, respectively. Predictions of the sine to triangular waveform NLT relative to polarization voltages of 3.6 V and 5 V obtained with optimal parameters through stochastic dynamics in panels **h.** and **j.**,

respectively ($[N, \Theta]$ in panels h and j are [10,9] and [13,14], respectively). Colormaps showing task performances as a function of N and Θ parameters for polarization voltages of 3.6 V and 5 V in panels **i.** and **k.**, respectively. Colormaps and predictions in panels d-k refers to results obtained by stimulating the network with a sine wave input with amplitude of 50 mV. Results shows that the effect of noise in the sine to triangular wave transformation is less detrimental compared to other NLT tasks.

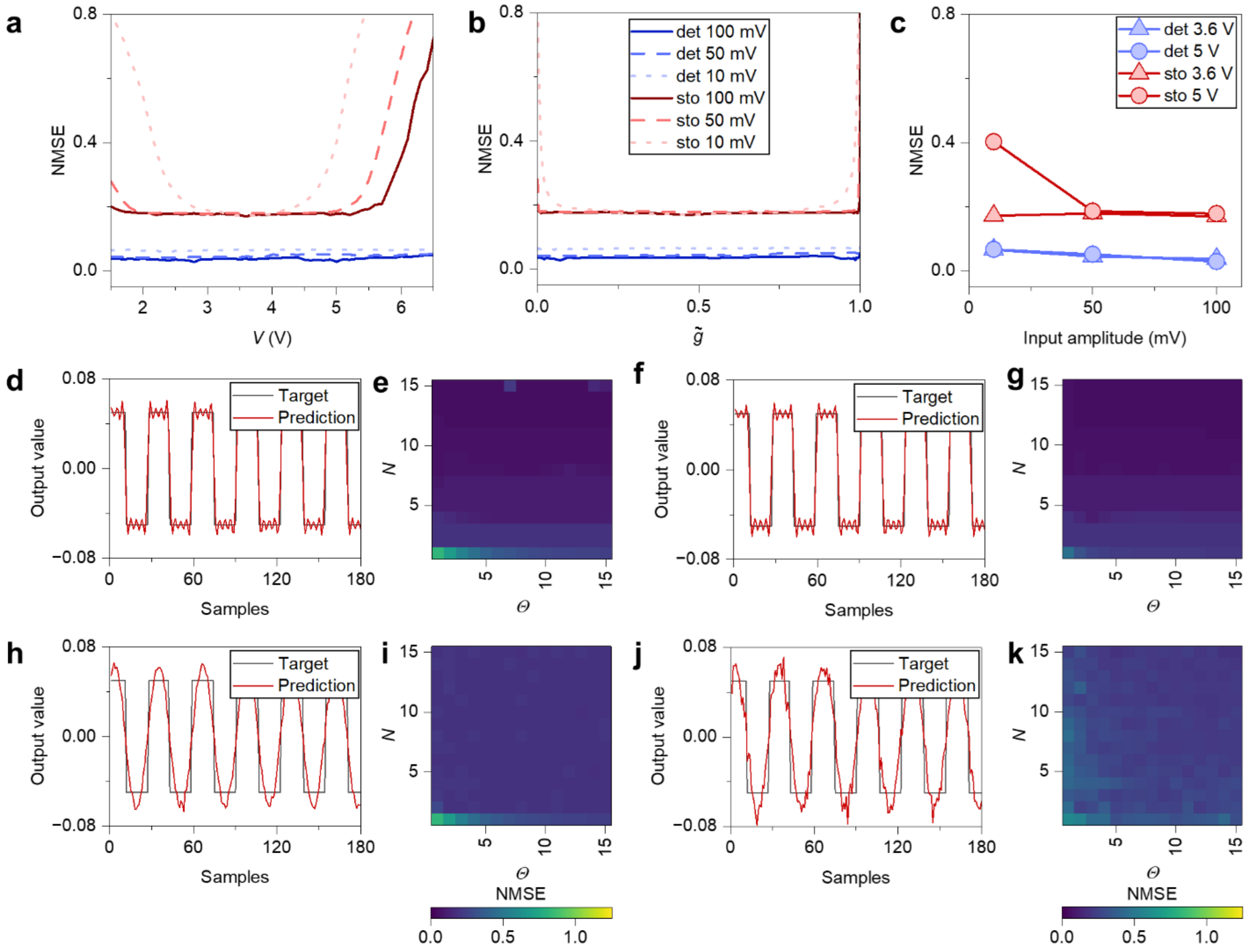

Supplementary Figure 17 | Nonlinear transformation (NLT) task. Simulation results of the sine to square waveform NLT task in terms of NMSE as a function of **a.** the bias voltage and **b.** steady state $\tilde{g}$ by considering deterministic and stochastic dynamics and for various amplitudes of the input signal. **c.** NMSE as a function of the input signal amplitude for deterministic and stochastic dynamics, by considering polarization voltages of 3.6 V ($\tilde{g}$ ~ 0.5) and 5 V ($\tilde{g}$ ~ 0.99). Predictions of the sine to square waveform NLT relative to polarization voltages of 3.6 V and 5 V obtained with optimal parameters through deterministic dynamics in panels **d.** and **f.**, respectively ($[N, \Theta]$ in panels d and f are [15,12] and [13,15], respectively). Colormaps showing task performances as a function of N and Θ parameters for polarization voltages of 3.6 V and 5 V in panels **e.** and **g.**, respectively. Predictions of the sine to square waveform NLT relative to polarization voltages of 3.6 V and 5 V obtained with optimal parameters through stochastic dynamics in panels **h.** and **j.**, respectively ($[N, \Theta]$ in panels h

and j are [10,13] and [15,15], respectively). Colormaps showing task performances as a function of N and Θ parameters for polarization voltages of 3.6 V and 5 V in panels **i.** and **k.**, respectively. Colormaps and predictions in panels d-k refers to results obtained by stimulating the network with a sine wave input with amplitude of 50 mV. Here, it is possible to observe that the effect of noise is to smooth transitions between minimum and maximum values.

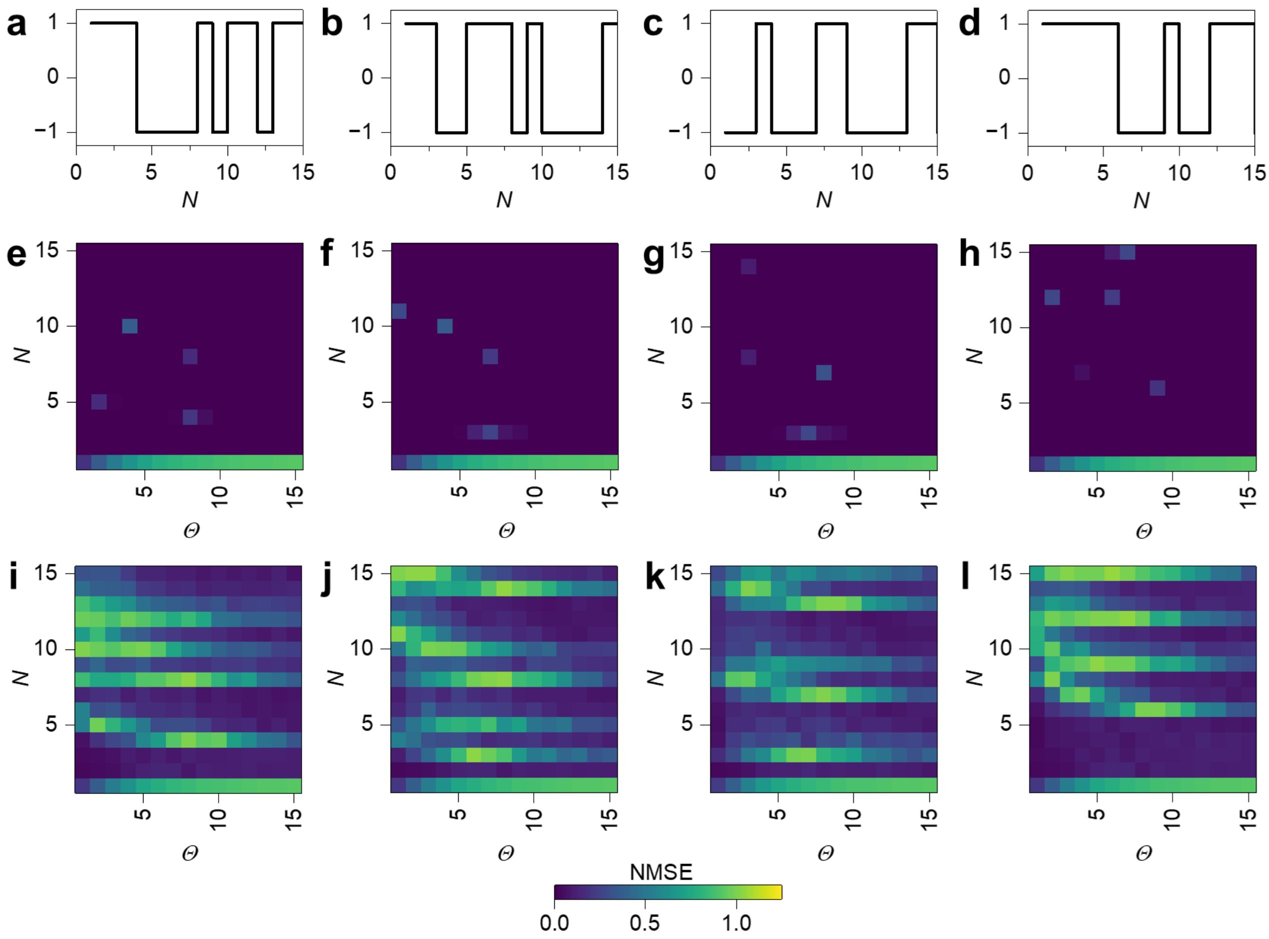

Supplementary Figure 18 | Effect of the masking scheme during time multiplexing on the sine to cosine waveform NLT task performance. a-d. Different masking schemes and corresponding colormaps showing task performances as a function of N and Θ parameters simulated by exploiting the **e-h.** deterministic and **i-l** stochastic model. Results have been obtained with a polarization voltage of 3.6, operating the network at $\tilde{g} \sim 0.5$. While the masking scheme does not substantially affect performance in terms of NMSE in the deterministic case, mask-dependent degradation of performances can be observed for some specific sets of $[N, \Theta]$ parameters. This indicates that the peculiar dynamics of the network induced by the masking scheme combined with the signal input can lead to computing performances of the system that can be less or more affected by stochastic effects.

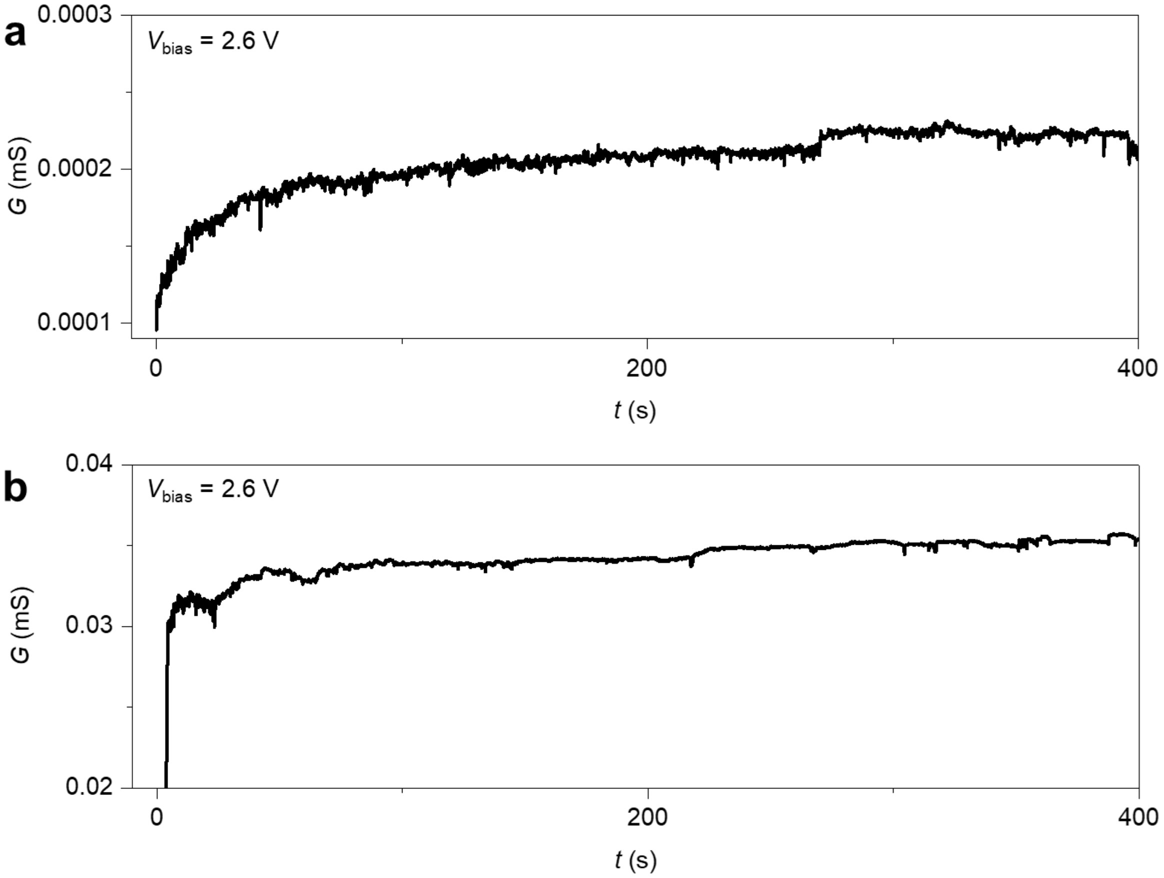

Supplementary Figure 19 | Tuning the steady state by network density. Example of the dependence of the steady state on the network density under the same bias stimulation, where the conductance time trace of the network realized by drop casting Ag NWs in isopropanol solution with a concentration of ~ 0.09 mg ml^{-1} (panel a) is compared with the one realized with a higher concentration of ~ 0.13 mg ml^{-1} (panel b). As expected, for the same applied voltage bias, the higher density network results in a steady state with higher conductance.

Supplementary Note 1 | Experimental characterization of network dynamics

The spatially distributed network activity can be accessed through characterization techniques that allows to measure local electrical properties of the networks and their evolution over time. For this purpose, techniques such as Electrical Resistance Tomography (ERT), voltage-contrast SEM imaging, conductive AFM, lock-in thermography, and multiterminal measurements can be exploited, as detailed in the following.

Electrical Resistance Tomography (ERT).[2,3] This quantitative and non-scanning technique allows reconstruction of the spatial distribution of conductivity across the network from boundary electrical measurements. Despite this technique enables to map the conductivity of networks over large areas (≈ 1 × 1 cm^2), it has a very low spatial resolution (≈ 2 mm) that does not allow to investigate how local effects in NW junctions reflects in the resulting network behavior. Furthermore, the long acquisition time required for mapping (≈ 40 s) does not allow to investigate the origin of conductance fluctuations over time in the network with high temporal resolution.

Voltage-contrast Scanning Electron Microscopy (SEM) imaging.[4,5] This scanning technique enables to acquire passive voltage contrast images that allow the observation of current pathways thorugh the network. Even if this technique endows high spatial resolution (estimated as ≈ 10 nm), the scanning area is limited (up to ≈ 100 × 100 μm^2). Since the resolution decrease while increasing the scanning area, this method does not allow to investigate the behavior of networks with more than few NWs with single junction resolution. Also, it should be highlighted that SEM imaging usually requires vacuum conditions that can alter the resistive switching mechanism. Despite advancements in low-vacuum SEM systems, voltage-contrast imaging to observe current pathways in self-organizing networks of nano objects still have to be demonstrated in air at ambient pressure.

Conductive Atomic Force Microscopy (C-AFM).[5] This scanning technique enables to measure local current flowing between the C-AFM tip and a reference electrode, allowing to evaluate the conductivity of specific network areas. Despite the high resolution of this technique (≈ few nm), the scanning area is limited (≈ 50 × 50 μm^2). Furthermore, the long acquisition time of this scanning

technique (≈ min) does not provide the temporal resolution for investigating physicochemical phenomena underlying conductance fluctuations observed in NW networks.

Lock-in thermography.[6] This technique enables to evaluate the local infrared emission (that can be converted to temperature) of the network when electrically stimulated, allowing to observe current pathways thorugh the network with a spatial resolution of ≈ 3 μm^2 over a scanning area of ≈ 1 × 1 mm^2. However, the state-of-the-art of this technique does not allow to investigate the dynamic behavior of the system due to the long acquisition time (≈ 50 s). Also, it should be highlighted that in this case the measurand is represented by the photons emitted by the network that are then used to infer local electrical properties, enabling to visualize only main conductive pahways that dissipate more power. In any case, advancements in the resolution of this technique can provide information on how power is dissipated through the network, an aspect that is directly related to the impact of network areas on the resulting network behavior.

Multiterminal measurements.[7–12] Multiterminal measurements have been widely exploited in NW networks mainly to nonlinearly map an input pattern in an output signal in the context of reservoir computing.[7–11] In all these contexts, the network was treated as black-box for processing an input signal for computing purposes. Recently, it has been shown that a multiterminal approach enabling synchronous probing of local electrical activity (16 electrodes over a sample of ≈ 1 × 1 cm^2) can be exploited to extract information on the local network activity and on its impact on the resulting network behavior.[12] By synchronously measuring the voltage over floating network distributed across the network and by reconstructing voltage maps and conductance matrices, it has been shown that multiterminal measurements can be exploited to monitor the spatial distribution of nonlinear activity, thus enabling to observe the impact of network areas on the resulting network behavior. Despite further advancements in multiterminal characterization are required to enhance the spatial resolution of this technique (for this purpose, multiterminal setups with an increased number of probing electrodes are required), this represents a promising technique for investigating how the resulting network behavior arises from local network activity under electrical stimulation.

Supplementary References

1. Gardiner, C. W. *Handbook of Stochastic Methods - For Physics, Chem, Nat. Sciences*. (Berlin, 1986).
2. Milano, G., Cultrera, A., Boarino, L., Callegaro, L. & Ricciardi, C. Tomography of memory engrams in self-organizing nanowire connectomes. *Nat Commun* **14**, 5723 (2023).
3. Milano, G. *et al.* Mapping Time-Dependent Conductivity of Metallic Nanowire Networks by Electrical Resistance Tomography toward Transparent Conductive Materials. *ACS Appl Nano Mater* acsanm.0c02204 (2020) doi:10.1021/acsanm.0c02204.
4. Manning, H. G. *et al.* Emergence of winner-takes-all connectivity paths in random nanowire networks. *Nat Commun* **9**, 3219 (2018).
5. Nirmalraj, P. N. *et al.* Manipulating Connectivity and Electrical Conductivity in Metallic Nanowire Networks. *Nano Lett* **12**, 5966–5971 (2012).
6. Li, Q. *et al.* Dynamic Electrical Pathway Tuning in Neuromorphic Nanowire Networks. *Adv Funct Mater* **2003679**, 2003679 (2020).
7. Sillin, H. O. *et al.* A theoretical and experimental study of neuromorphic atomic switch networks for reservoir computing. *Nanotechnology* **24**, (2013).
8. Avizienis, A. V. *et al.* Neuromorphic atomic switch networks. *PLoS One* **7**, (2012).
9. Demis, E. C. *et al.* Atomic switch networks—nanoarchitectonic design of a complex system for natural computing. *Nanotechnology* **26**, 204003 (2015).
10. Lilak, S. *et al.* Spoken Digit Classification by In-Materio Reservoir Computing With Neuromorphic Atomic Switch Networks. *Frontiers in Nanotechnology* **3**, 1–11 (2021).
11. Diaz-Alvarez, A., Higuchi, R., Li, Q., Shingaya, Y. & Nakayama, T. Associative routing through neuromorphic nanowire networks. *AIP Adv* **10**, (2020).
12. Pilati, D., Michieletti, F., Cultrera, A., Ricciardi, C. & Milano, G. Emerging Spatiotemporal Dynamics in Multiterminal Neuromorphic Nanowire Networks Through Conductance Matrices and Voltage Maps. *Adv Electron Mater* (2024) doi:10.1002/aelm.202400750.
